# Congo Red and amyloids: history and relationship

**DOI:** 10.1042/BSR20181415

**Published:** 2019-01-15

**Authors:** Elmira I. Yakupova, Liya G. Bobyleva, Ivan M. Vikhlyantsev, Alexander G. Bobylev

**Affiliations:** 1 Institute of Theoretical and Experimental Biophysics, Russian Academy of Sciences, Pushchino, Moscow Region 142290, Russia; 2 Pushchino State Institute of Natural Sciences, Faculty of Biophysics and Biomedicine, Pushchino, Moscow Region 142290, Russia

**Keywords:** amyloid dye, amyloid staining, amyloids, amyloid detection, amyloidosis, Congo red

## Abstract

Staining with Congo Red (CR) is a qualitative method used for the identification of amyloids *in vitro* and in tissue sections. However, the drawbacks and artefacts obtained when using this dye can be found both *in vitro* and *in vivo*. Analysis of scientific data from previous studies shows that CR staining alone is not sufficient for confirmation of the amyloid nature of protein aggregates *in vitro* or for diagnosis of amyloidosis in tissue sections. In the present paper, we describe the characteristics and limitations of other methods used for amyloid studies. Our historical review on the use of CR staining for amyloid studies may provide insight into the pitfalls and caveats related to this technique for researchers considering using this dye.

## Introduction

In amyloidosis diseases, amyloids can be detected in the kidney, liver, brain and other human and animal organs. The development of neurodegenerative human diseases, such as Alzheimer’s disease and Parkinson’s disease, is thought to be the consequence of accumulation of insoluble amyloid plaques in the nerve tissue [1]. However, amyloids have been observed serving biological functions beyond the development of pathological processes. Since the beginning of the 21st century, researchers have reported evidence that amyloids form in the body to perform specific functional roles [2]. These so-called ‘functional amyloids’ have been observed in both prokaryotes [3–5] and eukaryotes [2,6–8]. For instance, in *Escherichia coli*, amyloids contribute to the formation of biofilms [5]. Amyloids are also involved in melanin formation in the melanosomes of human skin cells [2].

The study of the amyloid formation process is currently one of the most important tasks in this field. Research in this area involves not only understanding how amyloidosis develops and how to prevent amyloid plaque formation but also investigating functional amyloids.

Staining with Congo Red dye (CR) is one of the major methods used to detect the amyloid structure of protein aggregates. However, a series of experiments have shown that CR staining is insufficient for confirmation of the amyloid nature of protein aggregates. This review details the history of amyloid research using CR dye. Some drawbacks and artefacts obtained when using this dye are described.

## Discovery and study of amyloids

In 1814, Colin and Gaultier de Claubry identified the blue staining reaction of starch with iodine and sulphuric acid [9]. One of the founders of the cell theory, Matthias Schleiden (1804–1881), who was interested in studying the chemical composition and anatomical structure of plants, proposed application of the iodine-sulphuric acid test for the detection of starch in plants (1838) (cited in [10]). In his later works, Schleiden wrote that starch formation occurred in plants, and the term ‘amyloid’ was introduced for the description of ‘a normal amylaceous constituent of plants’ [9].

Rudolph Virchow employed the term ‘amyloid’ in 1854 when he described the peculiar reaction of the corpora amylacea in the nervous system with iodine [9]. Interestingly, it is now known that these corpora are not amyloids [9]. Virchow suggested that cerebral corpora amylacea are similar to carbohydrates in nature and form deposits during ‘lardaceous’ or ‘waxy’ changes in the liver [9]. In 1859, Schmidt [11] denied this opinion, reporting a high proportion of nitrogen in organs infiltrated by amyloids. In the same year, Friedreich and Kekule [12] showed that waxy spleen tissue contained no material that corresponded chemically to amylon (that is, starch) or cellulose. These were the first steps toward investigation of the nature of amyloid deposits, which had been observed in organs since the 17th century.

The first mention of amyloidosis was most likely made by Fonteyn in 1639 [9,13]. He described an enlarged human spleen filled with sizable white inclusions. It is currently assumed that these inclusions were amyloid in nature [9,13]. In 1789, Antoine Portal was the first to describe liver amyloidosis [9]. Consequently, the term ‘amyloidosis’ encompasses a group of diseases associated with amyloid deposits in tissues and organs.

In 1842, Carl Rokitansky reported that the livers of patients with tuberculosis or syphilis became enlarged after infiltration by a grey, albuminous, gelatinous substance [9]. It appears that he was the first person to state that lardaceous/amyloid deposits occurred in cases of tuberculosis, syphilis and mercury poisoning [9].

In 1856, Samuel Wilks studied lardaceous viscera in elderly patients and concluded that the observed changes in organ tissue were not related to syphilis or tuberculosis. In 1865, Wilks found similar changes in the liver, kidney and adrenal glands in patients without diseases such as syphilis, tuberculosis or bony disease [9]. In 1867, Weber detected amyloidosis in a patient with multiple myeloma. In this patient, amyloids were found in the left ventricle of the hypertrophied heart, the kidneys and the spleen [9].

Further investigation of amyloidoses continued with the application of histopathological dyes, such as CR (1922) [14] and thioflavin (1959) [15], which were used instead of iodine. Until 1959, a histochemical approach was the common method for detecting amyloid deposits.

Structural investigations of amyloids began in the 1930s with the use of X-ray diffraction [16]. In 1935, Astbury and Dickinson, in the paper ‘The X-ray interpretation of denaturation and the structure of the seed globulins’ based on the process of egg albumin denaturation, concluded that ‘heat-denaturation of the albumins, for instance, merely makes the X-ray photograph more like that of a random arrangement of fibres of β-keratin’ [16]. Hence, Astbury and Dickinson [16] first noted the distinctive X-ray fibre diffraction pattern later called ‘cross-β’. With active use of the X-ray diffraction method, it was concluded that amyloids are composed of polypeptide chains extended in the so-called cross-β conformation [17,18]. In the cross-β structure, the individual strands of each β-sheet run perpendicular to the fibril axis (4.7 Å spacing) whereas the β-sheet (∼10 Å spacing) are parallel to the fibril axis [16,19].

In 1959, Cohen and Calkins [20] undertook electron microscopic studies of amyloid tissues in rat kidneys and human kidneys and skin. They observed amyloid deposits with a fibrillar structure ∼75–140 Å wide and ∼1000–16000 Å long [20]. The authors supposed that a challenge in protein detection within the deposits is the insolubility of amyloids in organic solvents [20].

In 1997, atomic force microscopy, cryoelectron microscopy [21,22] and solid-state NMR spectroscopy [23,24] were employed for investigations of amyloid morphology. Researchers began to elucidate the morphology of amyloid protofilaments, protofibrils and fibrils [26–28]. It was shown that the same protein can form amyloid fibrils, which can have different morphologies, such as coiled and ribbon-like fibrils of Аβ-peptide [25]. It was later found that amyloids may form not only structured fibrils but also amorphous aggregates [28–31].

Research focused on amyloids includes the study of prions, which were discovered by Prusiner in the 1980s [32]. Prions are a unique class of infectious agents where proteins are the basis of infectivity. In mammals, prions cause fatal neurodegenerative diseases, such as Creutzfeldt–Jakob disease in humans, sheep scrapie and bovine spongiform encephalopathy [33,34]. All of these diseases are caused by the PrP protein, which has a conformationally altered form (PrPSc) that can convert the normal host-encoded protein (PrPC) into this altered form [35]. The resulting altered form is the amyloid cross-β conformation.

In addition to the discovery of amyloids and prions that are responsible for the development of diseases, functional amyloids have also been discovered [2–8]. Since the beginning of the 21st century, data on protein aggregates with a cross-β structure that do not cause development of a pathological process but play a specific role in the organism have been reported. Functional amyloids have been observed in both prokaryotes [3–5] and eukaryotes [2,6–8]. It has been shown that in humans, functional amyloid fibrils are formed from proteolytic fragments of the Pmel17 protein in melanosomes [2]. These fibrils are involved in the polymerisation of melanin precursor into melanin and play a cytoprotective role by sequestering toxic intermediates produced during melanin synthesis and/or by templating and accelerating melanin production [2]. It was also suggested that amyloids play an essential role in the formation of long-term memory in animals. This idea is based on a study showing that the translational regulator CPEB of *Aplysia californica*, with prion-like properties, plays a key role in long-term synaptic changes [36].

In recent studies, it has been reported that many proteins, under certain *in vitro* conditions, are able to form amyloid-like aggregates or fibrils [37]. However, it is known that not all of these proteins form amyloids in humans or animals [38]. The term ‘amylome’ has been introduced to describe the universe of proteins that can potentially generate amyloid-like fibrils [38].

Today, there are multiple methods available for the study of amyloids (Table 1). With these methods, it is possible to detect amyloids *in vitro* and *in vivo*, to determine their structure and morphology in tissue sections and *in vitro*, and to explore the aggregation kinetics of amyloidogenic proteins (Table 1).

**Table 1:** Methods used to study amyloids *in vitro* and *in vivo*

Method	Characteristics and peculiarities	Applicability				Limitations
		Detection of amyloids	Study of amyloid structure	Study of amyloid morphology	Study of aggregation kinetics	
**Immunohistochemistry and Immunochemistry**	Immunohistochemistry is applied in pathology to visualise and localise protein aggregates and inclusions found in tissue sections of individuals with amyloidosis [39]. This technique is widely available and easily applicable in most pathology laboratories [40]. Immunohistochemistry uses antibodies for detection of amyloids. The availability of novel monoclonal antibodies targeting amyloidogenic precursors [41,42] and antibodies is directed against the amyloid fibrils [41,43]. Conformation-specific antibodies recognise soluble oligomers [44,45] or fibrils from many types of amyloid proteins [46–49], regardless of sequence. A small, bispecific antibody-based radioligand capable of crossing the blood–brain barrier can bind to intracerebral Aβ, allowing for *in vivo* visualisation [50]. This technique also uses luminescent–conjugated oligothiophenes as a unique class of amyloid dyes [51,52,53].	Yes	No	No	No	Commercially available antibodies for various proteins are often suboptimal for the identification of the same proteins in an amyloid fibril conformation [54]. This is caused by the proteins adopting different conformations and a variety of modifications during fibril formation, such as N- or C-terminal truncation [54]. To overcome this limitation, novel conformation-specific antibodies were designed [44–47]. At present, they are not yet widely used and require additional testing. Novel techniques that use luminescent–conjugated oligothiophenes [51–53] and the antibody-based radioligand [50] also need to be tested further. These novel developments are primarily used to detect Aβ fibrils and occasionally α-synuclein fibrils [46].
**Staining with CR**	The binding of CR to amyloids *in vitro* induces a characteristic increase in CR absorption leading to a red shift of its absorbance peak from 490 to 512 nm and the presence of a unique shoulder peak at approximately 540 nm [55,56]. Amyloid is detected by the increased optical anisotropy after CR binding [57], which is called the ‘apple-green birefringence’ (under crossed polarisers) [58]. ‘Apple-green birefringence’ is used to detect amyloid deposits in tissues and in *in vitro* studies of amyloids [15,57,58]. Today, this method is commonly used in histopathology laboratories because it is simple and cost-effective. There are different modifications of the method, including CR fluorescence (CRF) [59,60].	Yes	No	No	No	There exists some limitations (additional information is given in the main text).
**Staining with Thioflavin Т/S**	Thioflavin fluorescence is a classical method for detecting and analysing amyloids in tissue samples [61]. Thioflavin T is selectively localised to amyloid deposits, thereupon exhibiting a dramatic increase in fluorescent brightness [61]. This staining is also used in *in vitro* studies [62,63]. Upon binding to amyloid fibrils, Thioflavin T gives a strong fluorescence signal at approximately 482 nm when excited at 450 nm [64]. The fluorescence intensity scales linearly with amyloid fibril mass (e.g., with the number of available binding sites). Based on increasing fibril mass, the concentration of amyloid is calculated [65]. Reductions in Thioflavin T emission intensity are often interpreted as an indicator of fibril growth inhibition [66].	Yes	No	No	Yes	Staining with Thioflavin T/S is easy to perform, but the requirement of fluorescence microscopy limits the usefulness of this staining method [61]. Other tissues, such as cartilage, elastic fibres and mucopolysaccharides, can also be stained with ThT [67–69]. Sometimes, even at substoichiometric dye/monomer ratios, ThT undergoes substantial self-quenching that results in a non-linear relation between its binding and emission properties [63]. In this regard, without preliminary experiments, the use of this method for the study of fibril formation kinetics is problematic. ThT fluorescence is not generally a suitable tool for the detection of oligomeric intermediates during amyloid fibril growth [62]. Nevertheless, this dye binds to some oligomers [70–72].
**Circular dichroism**	This method is used to determine the secondary structures of proteins and peptides in amyloid aggregates [73,74]. This technique operates by the differential absorption of the left- and right-handed components of circularly polarised light by chiral molecules in solution [75].	No	Yes	No	No	This technique can be used only for determination of changes in secondary structure and is sensitive to aromatic compounds present in the sample. It can be employed only in *in vitro* studies.
**Fourier transform IR spectroscopy**	Fourier transform IR spectroscopy is an absorption spectroscopy in which the transitions detected are those arising from vibrational modes of bonds involving heteroatoms [77,78]. The presence and relative abundance of β-sheet structure in peptides and proteins can be assessed by this technique [76] also for amyloid studies [79,80]. Methodologies exist to acquire spectra from proteins in any physical state, including crystals, powders, thin films, and aqueous solutions, as well as from membrane-bound proteins [76].	No	Yes	No	No	There is water interference [76]. High protein concentration is necessary [76]. This technique can be employed only in *in vitro* studies.
**NMR**	Molecular and supramolecular structures of amyloid fibrils can be probed by various solid-state NMR techniques [24,82–89].	No	Yes	Yes	Yes	This technique is expensive and difficult [81]. Analysis is limited to small proteins [81]. It can be employed only in *in vitro* studies.
**X-ray diffraction**	The method is used to examine the structures of insoluble amyloid fibres [90,91]. With this method, it is possible to reveal the presence of the cross β-sheet structure by detection of the 4–5 Å equatorial and 10–12 Å axial reflections that correspond to the inter-chain distance and to the face-to-face separation of β-sheets, respectively [92,93].	No	Yes	No	No	This method can only be used when the sample is in the crystallised form. It is difficult to employ this method to study intermediates at the initial aggregation stages. This technique can be employed only in *in vitro* studies.
**Small-angle X-ray scattering**	This technique can be used to investigate the structure, folding and conformational dynamics of globular proteins, including multidomain and multisubunit proteins [94]. Small-angle X-ray scattering is a powerful and flexible technique for the characterisation of structural variations in amyloid fibrils [95–98].	No	Yes	Yes	No	This technique is expensive and difficult. This technique can be employed only in *in vitro* studies.
**Cryoelectron microscopy**	Cryoelectron microscopy technology allows for high resolution (less than 5 Å) determination of the atomic structures of amyloid fibrils *in vitro* [99–101]. Also in this technique, it is possible to explore amyloids extracted from tissues (*ex vivo*) [102,103].	No	Yes	Yes	No	It is difficult or impossible to study amorphous amyloid aggregates using this technique. The process of sample preparation is very complicated.
**TEM**	Since amyloid fibrils have unique electron microscopy characteristics [104,105], electron microscopy is routinely used in the analysis of kidney biopsies in the United States and other countries [104]. All types of amyloid deposits seen in different tissues, are mainly composed of bundled, not branched, straight fibrils, ranging from 6 to 13 nm in diameter (average 7.5–10 nm) and 100–1600 nm in length [106]. However, other morphological characteristics can also exist [107]. To increase the diagnostic significance of the method, immunogold electron microscopy, a technique that combines immunohistochemistry with electron microscopy, can be used [108]. With this technique, it is possible to explore both amyloids extracted from tissues and amyloids generated *in vitro* [106].	Yes	No	Yes	No	In some cases, this technique is not suitable to diagnose amyloidosis. For instance, immunotactoid glomerulopathy and fibrillary glomerulonephritis can be misdiagnosed as immunoglobulin light and heavy chain amyloidosis [109].
**Atomic force microscopy**	This technique can be used to investigate the size and morphology of protein aggregates in solution [110]. This method is also used to probe intrinsic properties of amyloid fibrils, such as mechanical strength and Young’s modulus [111].	No	No	Yes	Yes	When contact mode is used, high shear forces cause damage to the fibrils and may require immobilisation strategies [112]. The tapping-mode in air requires a ‘dried’ sample, so it cannot probe fibril processes directly; as a result, potential artefacts from dehydration (e.g., salt crystals, fibril damage) may be observed, and the degree of hydration is not known and cannot be controlled [112]. For the tapping-mode in liquid, current scan speeds are too slow to image rapid processes, and often, samples (fibrils) need to be well-adhered to a surface. Other atomic force microscopic techniques, including the use of support surfaces (e.g., lipid bilayers) are often difficult to use [112].
**Dynamic light scattering**	Dynamic light scattering is a laser scattering technique capable of unbiased analysis of size distributions for diffusing particles in the nanometre to micrometre size range [113]. The ability to resolve multimodal size distributions and make absolute size measurements makes dynamic light scattering a powerful technique for systems with heterogeneous species. It has been used to quantitatively study fibril formation in a range of systems from different proteins [114–117].	No	No	No	Yes	A hydrodynamic radius of the particle is measured, but this can differ from the true radius. As a result, determination of the real size and shape of macromolecules is difficult.
**Fluorescence correlation spectroscopy**	This highly sensitive analytical technique is used to measure dynamic molecular parameters, such as diffusion time (from which particle size can be calculated), conformation and concentration of fluorescent molecules [118,119]. It has been particularly powerful for characterising size distributions in molecular associations (e.g., dimer/multimer and fibril formation) both in well-behaved thermodynamically equilibrated systems *in vitro* and in more complex environments *in vivo*. Fluorescence correlation spectroscopy could therefore be used as a highly sensitive and specific competition assay to identify potential inhibitors of fibril formation [118].	No	No	No	Yes	This technique requires the use of fluorescent tags to label amyloid samples. This makes it difficult and rarely used.
**Analytical size-exclusion chromatography**	The method is used to separate a diverse range of differently sized particles by passing a solution containing the particles through a partially permeable gel medium. It is used to analyse intermediates (mainly, oligomers) and identify soluble aggregates in tissue [120,121].	No	No	No	Yes	It is impossible to study aggregates with high molecular weight. This method is inefficient for scale-up because size-exclusion chromatography performs poorly on large liquid volumes.
**Analytical ultracentrifugation**	This technique is based on the sedimentation velocity analysis used to determine the size, shape, and hydrodynamic behaviour of soluble macromolecules, including amyloid fibrils, as well as to study the process of amyloid aggregation [122–125].	No	No	No	Yes	This technique is used only for specific tasks.

A considerable number of scientific studies on the structure and function of amyloids have been conducted since the beginning of the second half of the 19th century. During this time, staining with CR dye remained a conventional technique for determining the amyloid nature of protein aggregates.

## CR dye

Dyes are usually aromatic, heterocyclic compounds, some of which are toxic and possibly carcinogenic [126]. CR is a direct diazo dye that is intended primarily for the colouration of paper products. It is toxic and possibly carcinogenic and mutagenic [126–128]. CR is the sodium salt of benzidinediazo-bis-1-naphtylamine-4-sulphonic acid (formula: C_32_H_22_N_6_Na_2_O_6_S_2_; molecular weight: 696.66 g/mol; see the chemical structure in Table 2) [126]. The Colour Index Number of CR is 22120.

**Table 2:** Relation between different dyes and selective staining of amyloid (by [129] with our modifications)

Dye*	Amyloid	Collagen	Elastic fibres**	Cytoplasm
CR (C_32_H_22_N_6_Na_2_O_6_S_2_) 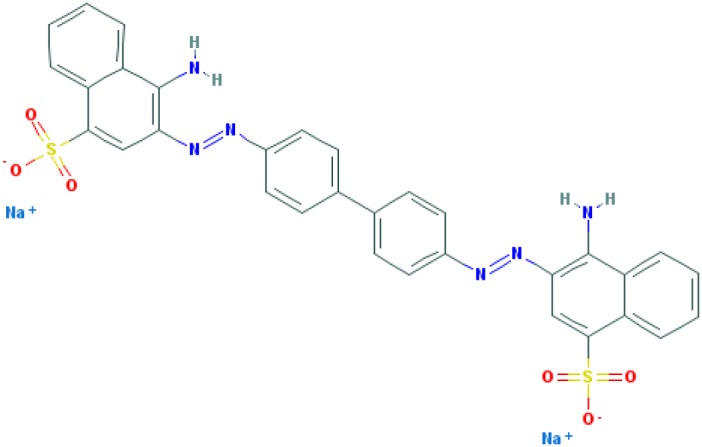	+++++	±	+++	±
Congo Corinth (C_32_H_23_N_5_NaO_7_S_2_^+^) 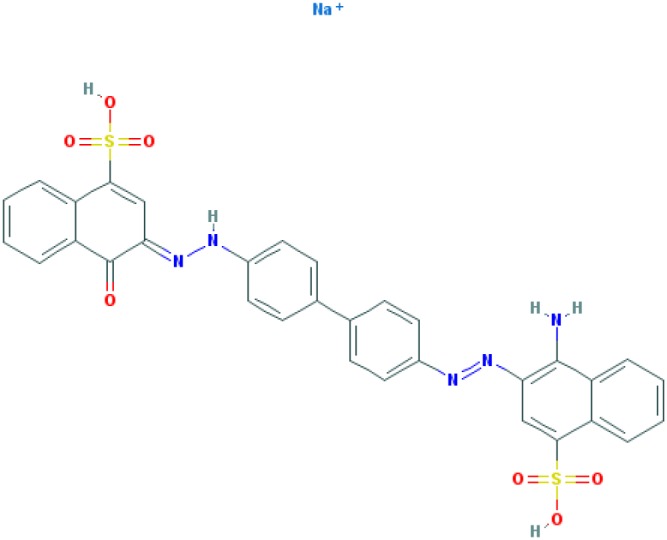	++++	±	+++	±
Benzopurpurin 4B (C_34_H_26_N_6_Na_2_O_6_S_2_) 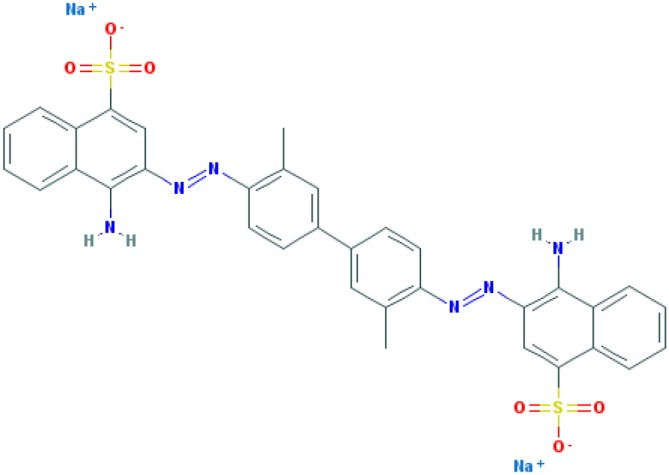	++++	0	++	0
Vital Red (C_34_H_25_N_6_Na_3_O_9_S_3_) 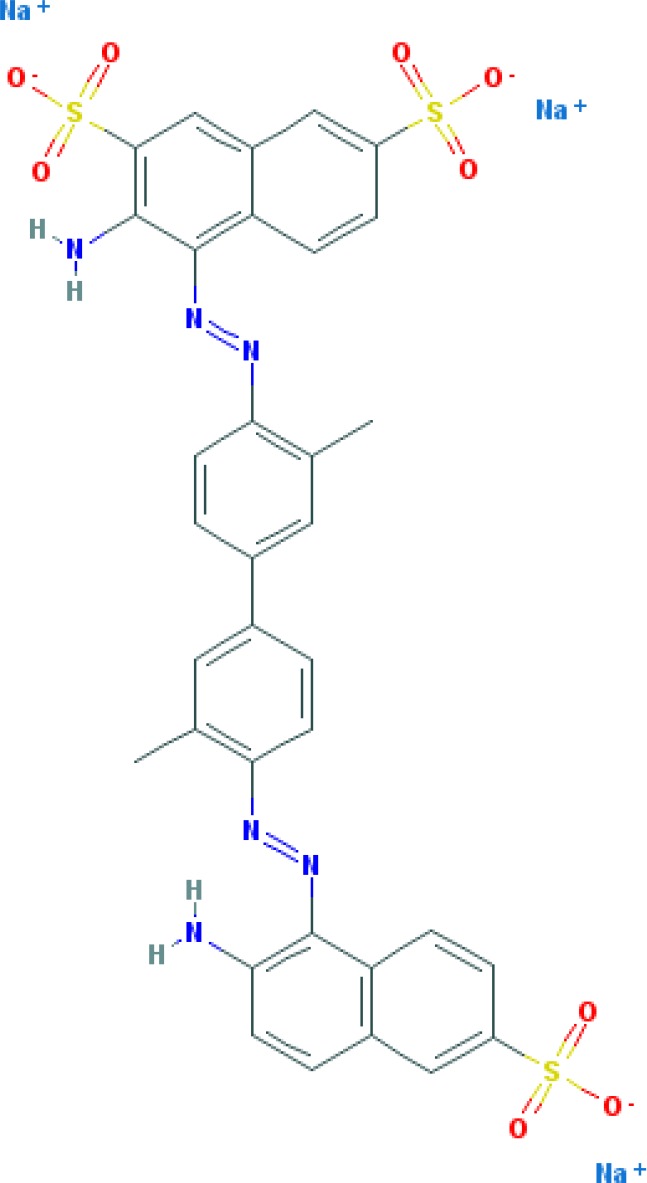	+++	0	+	0
Trypan Blue (C_34_H_24_N_6_Na_4_O_14_S_4_) 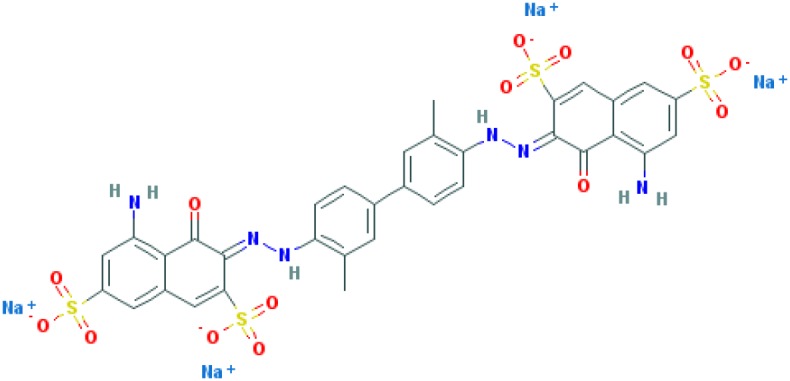	++	0	±	0
Amidoblack 10B (C_22_H_14_N_6_Na_2_O_9_S_2_) 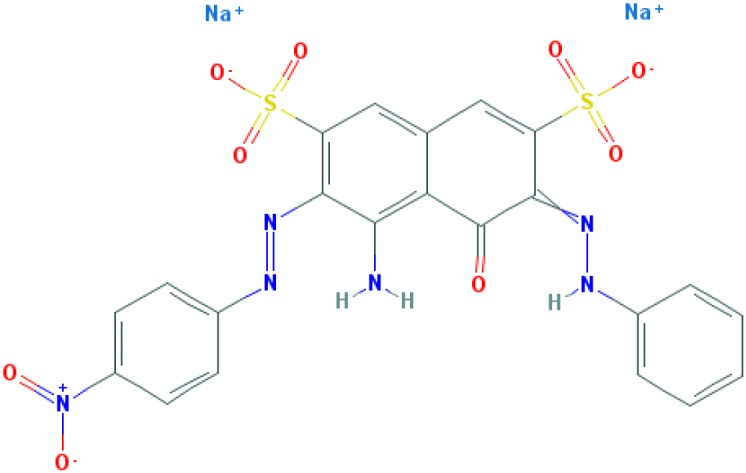	+	+	+	+
Biebrich Searlet WS (C_22_H_14_N_4_Na_2_O_7_S_2_) 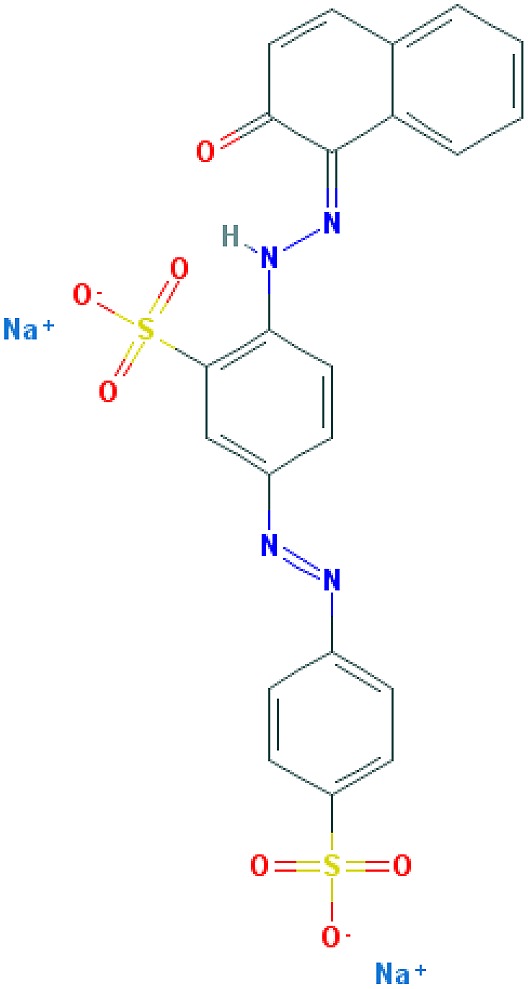	0	0	0	0
Aniline Blue WS (C_32_H_25_N_3_Na_2_O_9_S_3)_ 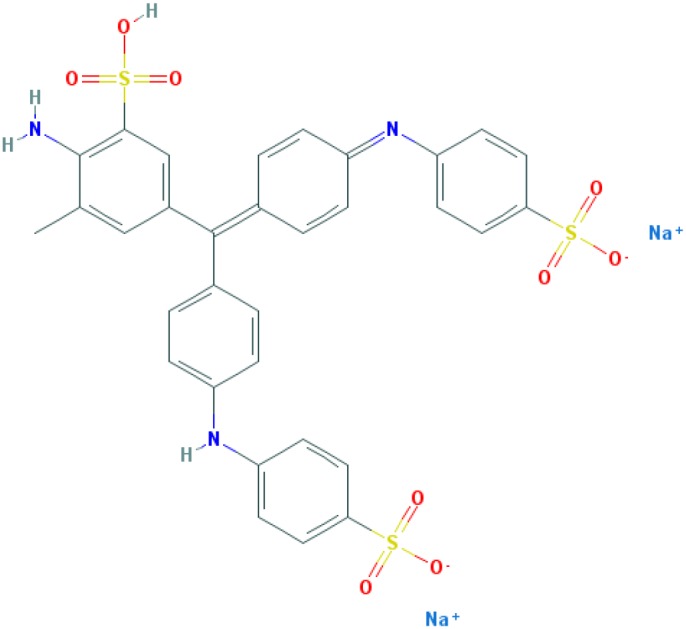	±	±	±	±
Acid Fuchsin (C_20_H_17_N_3_Na_2_O_9_S_3)_ 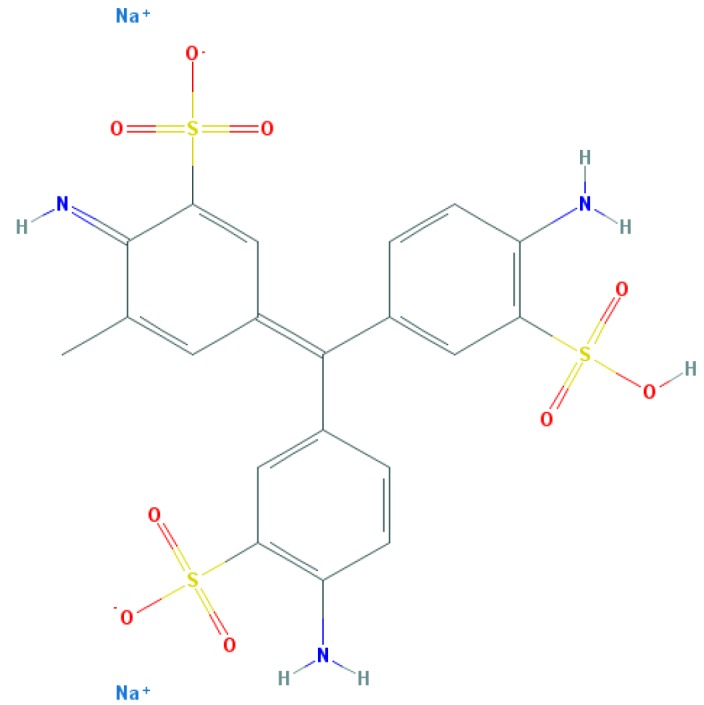	+++++	+++++	+++++	+++++

Tissue colouration degree: 0 denotes no colour, ± → + → ++ → +++ → ++++ → +++++ is from poor colour to rich colour.

* Formulas and pictures are taken from the Open Chemistry Database, PubChem.

** Membrana elastic interna of arteriae interlobares in kidney.

CR was discovered in 1883 by chemist Paul Böttiger when he tried to synthesise a substance that could be used as a pH indicator [130]. In 1885, this dye was named as CR [130]. When CR is present as a disodium salt in alkaline or weakly acidic solutions, a red colour is produced. In strongly acidic conditions, as the free acid, the maximum absorption moves to longer wavelengths in the yellow and orange regions, giving a violet or blue colour [129].

This dye became the first of many so-called ‘Congo’ direct dyes produced at the dyestuff chemical laboratory of the Friedrich Bayer Company in Elberfeld, Germany, where Böttiger worked [130]. After the discovery of CR, many textile dyes received the Congo name: Congo Rubine, Congo Corinth, Brilliant Congo, Congo Orange, Congo Brown and Congo Blue. The word ‘Congo’ in the dye name was used for marketing purposes [130]. Many aniline dyes were tested as possible histological dyes [131,132], which included testing for staining of amyloids (Table 2).

In 1886, CR was employed as a pH indicator to detect acid in the intestinal tracts of animals [133]. In 1922, Bennhold was the first to discover that CR can be used for identification of amyloids in organisms after injecting the dye into blood [14,134,135]. Since then, CR has been used for identification of amyloids both *in vivo* and *in vitro*.

## The use of CR in the study of amyloidosis

As mentioned above, the use of CR for amyloidosis identification began with experiments performed by Bennhold [14]. From the moment this new class of diseases, ‘amyloidoses’, was discovered, clinicians and scientists have searched for ways to diagnose these diseases and develop treatment methods. Bennhold described, for the first time, an approach for laboratory diagnosis of amyloidosis in 1923 [14]. He intravenously injected 10 ccs of a 1% solution of CR into 21 healthy subjects and 21 patients with different diseases [14]. In patients with nephrotic syndrome, a disappearance of CR in the blood occurred faster than in healthy individuals. In ten patients with amyloidosis secondary to pulmonary tuberculosis, clearance was apparently even more rapid, owing to an erroneous interpretation of the absence of CR in the urine. One of the patients with amyloidosis died 20 h after injection of the dye, and autopsy tissues were then obtained. The liver and spleen appeared to be stained by the dye. Microscopic studies of unstained frozen sections of the organs showed red areas [14]. This was the first microscopic demonstration of CR binding to amyloid [136]. Bennhold regarded the disappearance of 60% of the CR from circulating blood 1 h after injection as presumptive evidence of amyloid disease [134,135].

Subsequent studies concluded that the use of CR staining of amyloids also gives many false-positive results [141]. As a result, Bennhold’s method for diagnosis of amyloidosis was modified by many investigators including Friedman and Auerbach [137], Taran and Eckstein [138], Lipstein [139], Stemmerman and Auerbach [140] and others. The proposed modifications involved reducing the time between patient blood sampling and analysis and changing the criteria for clinically relevant dye content.

In 1948, Unger et al. [141], who criticised Bennhold’s CR test and its different modifications, pointed three significant mistakes made during the application of this method: the first lies in the assumption that the injected dye is completely mixed at the time of analysis; the second source of error lies in the assumption that little or no absorption of the injected CR takes place in amyloidosis at the reduced time (before 2- or 4-min specimens are obtained [138]); and the third source of error is the personal equation which enters into the colour matchings whenever optical colorimeters are used]. It should be noted that Unger et al. [141] also modified the CR test, calculating the theoretical initial concentration rather than using 2- or 4-min specimens for comparison and using 30 min rather than an hour as the end point [141].

In 1964, Stemmerman and Auerbach [140] reviewed the results of 649 cases of CR tests for amyloidosis performed on 446 patients, reported that the cases included 24.3% false negatives and 4.2% false positives and concluded that the chief cause of false-negative results was the minimal presence of amyloids and the principal reason for false-positive results was probably due to technical errors.

Notably, CR has been used for applications beyond detection of amyloidosis. Two years after Bennhold’s experiments (1925), Alder and Reimann [142] introduced the CR test as a reticuloendothelial system function test.

In 1976, Ouchi et al. [143] stated that there were no appreciable data on Bennhold’s test for the diagnosis of amyloidosis in scientific Japanese literature. This probably points to the fact that amyloidosis is an extremely rare disease in Japan [143]. Hence, it is clear why Japanese investigators explored other properties of CR. Japanese researchers presented data showing that intravenously injected CR was selectively taken up by Kupffer’s cells [144,145]. CR passes mainly through Kupffer’s cells and is later excreted into bile via hepatocytes; it also partially passes through other cells in the reticuloendothelial system [143–145].

Ouchi et al., while evaluating the CR test for its ability to detect amyloidosis of the liver, observed that the ‘CR test is not the best test for the diagnosis of amyloidosis’ [58]. No pathological features specifically influencing the CR index (CRI) were found by Japanese researchers during histological studies. At the same time, it was shown that the CRI is usually higher in liver diseases than in various other diseases [143]. Ouchi et al. [143] showed that an increase in the CRI is most visible in cases of liver cirrhosis and can also be explained by an obstructive change in liver blood flow followed by hepatic tissue damage. It should be noted that the authors did not provide any explanation for what CRI represented. At times, the authors used symbols (−/+/++/+++) as subjective estimates for the degrees of tissue colouration [146], and the results were occasionally given as percentages [143].

Although the CR test was recognised as suboptimal for the diagnosis of amyloidosis [143] and has been criticised [141], the development of CR-staining methods for the diagnosis of amyloidosis has continued to this day. Intravenous injection of CR, used in clinical practice for approximately 40 years, was replaced by staining of histological sections and biopsies with the dye [147].

It is commonly thought that CR-stained amyloid has an orange-red appearance under light microscopy and apple-green birefringence under polarised light during *in vitro* investigations and *in vivo* histological studies using tissue sections [9,147]. Several papers reported that this phenomenon was discovered by Divry (1927) and/or Divry and Florkin (1927) [148,149]. These authors described an increase in the intensity of the birefringence in CR-stained amyloid, but not a change in the colour of the specimen ([147]). Colours in CR-stained amyloid were first described by Ladewig (1945) [150] in his work ‘Double-refringence of the amyloid-Congo-red-complex in histological sections’. Green was not the only colour observed in his experiments; he stated that the colour ‘changes during rotation through 360° of the optical axes of the preparation twice from yellow to green, if conditions are optimal’ [150]. In this work, Ladewig also wrote about limitations when using CR: ‘its specificity is compromised by certain hyaline, mucous, fibrinoid or elastic tissue components also ‘taking’ CR’. The author suggested that this phenomenon is seen because other CR-positive, non-amyloid tissue components, such as certain hyalines, etc. ‘may be strongly anisotropic by themselves, a fact which is argument enough against their being regarded as amyloid’ [150].

Because of the geopolitical situation in Istanbul at the time Ladewig was conducting this research, he had no access to scientific literature on similar topics, and Howie and Brewer [147] noted that Ladewig’s investigations can be considered accurate and independent observations.

Ladewig was not the only investigator who described different colours under polarised light while studying CR-stained amyloids. Cooper [151] and Taylor et al. (1974) [152], who explored tissue sections with amyloid damage using CR staining, described that counter clockwise rotation of the analyser by 1-2 changes the colour to yellow-orange; a similar clockwise rotation produces a blue-green colour. Howie and Brewer stated in 2009 [147] that confusion regarding the origin of the term ‘apple-green birefringence’, which describes the change in colour when CR binds to amyloids in birefringence, began with a report by Hans-Peter Missmahl who was a physician in Tübingen under the direction of Bennhold (1920–2008). In 1957, Missmahl claimed that ‘Divry first noticed the increase in the strength of the birefringence of CR-stained amyloid. He was also struck by the appearance of a green polarisation colour’ (translated version from the German language [147]). Later, other researchers wrote that ‘Divry and Florkin were the first to observe that amyloid structures stained with CR, when observed in polarised light, as well as the increase in birefringence, lit up green’ (Diezel and Pfleiderer (1959) [153]). Wolman and Bubis (1965) [154], Benditt et al. (1970) [155] and others repeated similar incorrect explanations of the initial observation of apple-green birefringence in CR bound to amyloids (by [147]).

The phrase ‘apple-green birefringence’, often mentioned in articles on amyloid investigations, has apparently been used since 1972 [156]. This terminology probably originates from the word combination ‘apple-green immunofluorescence’, which was used in the 1950s. The use of ‘apple-green birefringence’ and ‘apple-green dichroism’ in textbooks and articles apparently promoted their subsequent citation [145]. Until now, observation of a green colour under polarised light is thought to indicate the presence of amyloids in CR-stained tissues. When this phenomenon was not observed, possible causes were considered, including inconsistencies in sample preparation. Specifically, it was shown that tissue sections <5 μm in thickness can produce false-positive results [15,61]. Conversely, other experiments showed that tissue thickness is not a significant factor in false-positive results and that the same area of a section or smear can appear green or yellow depending on the setup of the microscope [147].

Analysis of experimental data available before 2012 on CR apple-green birefringence has shown inconsistencies between the results of different studies [157]. In 160 scientific papers on staining of amyloids in tissue sections with CR, researchers reported only green birefringence or apple-green birefringence, even though only 31% of the illustrations showed a pure green colour [58,157]. In 66% of the papers, there were discrepancies between the descriptions of the colours reported in the text and the visual images shown in the figures [157]. These works mainly described discrepancies between reports of green-only and green-with-another-colour in the figures, or even between reports of green colour and total lack of green in the figures. Consequently, regardless of the colour observed, for example yellow/green and blue/green, the researchers described these colours as yellow or green. Combinations of yellow and blue colours are seen in practice more often than a pure green colour [147]. The various other colours, apart from the red that is seen when the polariser or analyser is progressively rotated from the crossed position, are also so-called ‘anomalous’ colours and are explained by the combination of absorption and changes in birefringence due to anomalous dispersion of the refractive index, usually with additional effects of strain birefringence in the optical system [147].

Howie and Owen-Casey [157] suggested that a regular use of the word ‘green’ in texts, papers, meetings and lectures provided the basis for the scientists to believe that observation of ‘green’ (including apple-green birefringence) is a reliable tool for diagnosis of amyloidosis [158].

Since 1965, CR fluorescence (CRF) has been used in research [59]. It is thought that this technique increases the recognition of tissue-bound CR by its property as a fluorochrome [159].

Nevertheless, until recently, CR has been used as a specific dye for amyloids. The question arises of how CR binds to substrate.

## The binding of CR to amyloids

CR was the first direct dye for cotton, which did not require a mordant or chemical to fix the colour to the material [130]. Cotton is composed of cellulose, the substance that constitutes most of the plant cell wall. It is known that the CR molecules align themselves along the linear molecules in cellulose and that hydrogen bonds form between these two substances [160].

**Figure 1:**
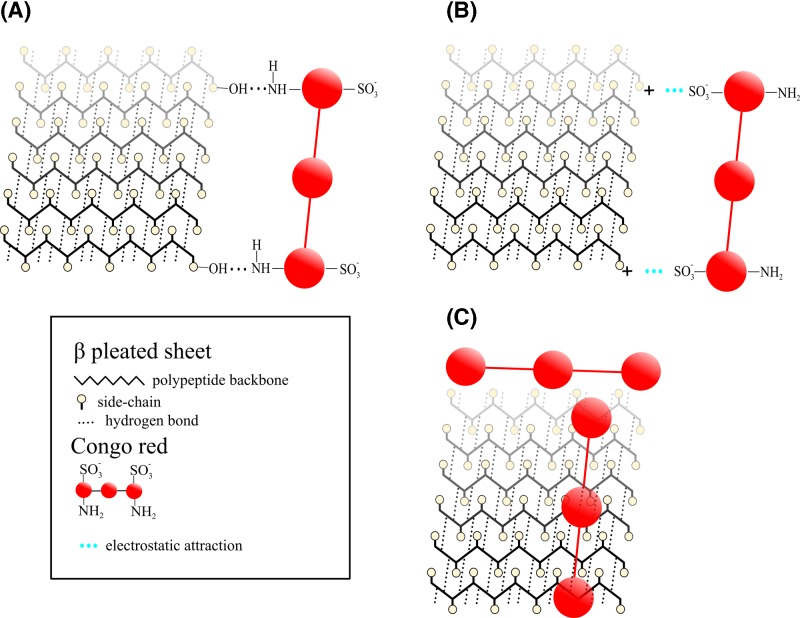
The main hypothetical models of the binding of CR to amyloids (**A**) Dye binding mediated by hydrogen bonding between primary hydroxyl groups of the peptide chain (similar to the polysaccharide chain) and the amino groups of CR (Puchtler et al., 1962 [132]). (**B**) CR molecule could bind to positively charged amino acid residues along the peptide chains (Klunk, W.E. et al., 1989 [161]). (**C**) Polar contacts drive CR binding (Reinke and Gestwicki, 2011 [166]).

Figure 1 presents the main concepts of the binding of CR to amyloids. Amyloids were discovered during histological studies of human tissues with the iodine-sulphuric acid test for starch [9]. Virchow, who visualised amyloids using this test, began to use the term ‘amyloid’, and he thought that their structure resembled cellulose rather than starch [9]. Considering this fact, it is not difficult to understand why the researchers assumed that the binding of CR to amyloids occurred in a similar manner as its binding to cellulose [132]. Puchtler et al. (1962) [132] believed that dye binding was mediated by hydrogen bonding between primary hydroxyl groups of the polysaccharide chain and the amino groups of CR (Figure 1A). They also showed that not all direct cotton dyes bind amyloid selectively [129]. Therefore, the mechanism of the binding of CR to amyloids may be different from the mechanism of cellulose staining mentioned above.

Cooper (1974) [151] suggested that CR may bind to amyloid by being partially entrapped and bound in ‘channels’ of the β-pleated sheet amyloid by non-specific close-range forces [161]. Still, this mechanism did not explain binding on the biochemical level.

In a paper by Klunk et al. in 1989 [161], it was shown that CR binding correlates well with the number of positively charged amino acids in a sample of amyloid fibrils (Figure 1B) [161].

The modern notion of CR binding to amyloids lies on the assumption that linear ligands can exploit the regular patterns (e.g., grooves) on the amyloid fibril surface in primary binding modes [162].

Molecular docking simulation results suggested that CR binds to sites parallel to the fibril axis (antiparallel to the β-sheets) [163] on amyloid fibrils [162–165] and protofibrils [162]. With the use of molecular docking simulations, a second site at the ‘end’ of the protofibril in an orientation antiparallel to the fibril axis and parallel to the β-sheets was also described (Figure 1C) [166]. Notably, this latter binding mode is represented only in approximately 11% of the total CR binding clusters, while the antiparallel orientation is clearly preferred (78% of the total binding) [166].

There is evidence demonstrating the binding of CR to proteins with different secondary structures [167]. This fact indicates that a specific amyloid structure is not mandatory for the binding of CR [167]. It has also been found that the binding of CR to protein molecules leads to protein oligomerisation [167]. Based on these data, the following conclusion on the possible mechanism of CR binding to proteins was made: ‘It is most likely that both the hydrophobic and the electrostatic components of the structure of CR are critical for its binding to proteins’ (2001) [167].

It is thought that the orientation of CR molecules on amyloid fibrils determines the dichroic and birefringent effects seen in tissue sections [168]. However, the physical basis of colours seen in CR-stained amyloid in polarised light was thoroughly described only in 2008 [168]. Birefringence indicates that a material has two refractive indices, depending on its orientation in polarised light. Usually, in studies on CR-stained amyloids, researchers report that experiments were carried out ‘under polarised light’. In most cases, this finding implies that examination of the sample was performed when the polariser and analyser were crossed [168]. In this case, the word combination ‘apple-green dichroism’ is used incorrectly [168]. Dichroism means that either a material has different amounts of absorption of light polarised in different planes, or the material varies from coloured to colourless, depending on the plane of polarisation. To explore this property, it is necessary to examine the material on a microscope fitted with either a polariser or an analyser, but not both, and when either the stage or the polarising filter is rotated, the appearance of the material should change from its deepest colour to colourless [169].

It has been shown that CR is dichroic [160,170] and, as a result, the change in intensity of red can be easy to see in smears of CR. Since amyloid fibrils are often haphazardly arranged, it is rather difficult to identify such a change in samples of CR-stained amyloid [168]. It has been shown that since the most typical property of CR-stained amyloid is the development of anomalous colours (yellow/green and blue/green) when using a crossed polariser and analyser setup, ‘apple-green birefringence’ should not be considered as the main feature to describe the properties of CR-stained amyloid [168].

When spectral methods were employed, characteristic pH-dependent changes in the CR absorption spectrum were defined. In strongly acidic conditions, CR has a maximum absorption that moves to longer wavelengths in the yellow and orange regions, giving a violet or blue colour [129]. This explains how CR can be used as a pH indicator, changing colour in the pH range 3–5 [172,173]. The peak absorption may also move to longer wavelengths when there is increased binding to a substrate, which is called the bathochromic shift [174]. The effects of pH upon binding may explain some reported differences in the optical properties of CR-stained amyloid and orientated CR, for instance in the wavelengths at which a property is at its maximum [147]. In addition, it has been found that the change in pH value of the medium leads to a change not only in colour but also in the solubility of CR [172].

In the 1970–80s, it was shown in stained tissue sections that observed acid-induced colour changes may be affected by the type of dye–substrate binding [156,177–179]. New modifications of CR staining methods were made to improve staining of tissue sections, however none of these procedures abolished concomitant staining of elastic fibres [171]. The researchers believed that staining of elastic fibres and amyloids was a result of non-ionic dye-binding forces [175,176]. However, studies that used a variety of different solutions and dye bath modifications designed to inhibit ionic, hydrogen or hydrophobic bonding did not show abolished staining of these two tissue substrates [177].

The authors of these studies concluded that the colour distinction between different stained substrates may reflect the dye indicator response to the acidic or basic nature of the substrate [171]. It appears that acidic and neutral substrates stained by CR display the colour induced by the acidic buffer, whereas basic substrates apparently accept protons from the cationic dye and, therefore, retain red colouration. In the case of amyloids, the red colouration observed in acidic conditions suggests the presence of basic groups [171]. According to these findings, the red colour of amyloids that develops after staining with CR might be a consequence of the presence of a comparatively large proportion of basic amino acids. However, this assumption does not explain the specific blue staining of collagen by CR, although this protein also has a large proportion of basic amino acids [178]. One explanation for this difference may be the variation in the positions of the basic amino acids within the protein and their availability in relationship to the dye molecules [171]. Nonetheless, removal of the ionisable basic groups in tissue by nitrous acid deamination resulted in minimal changes in the colour intensity of the red stain [171].

We next consider the known exceptions to the rules.

## Exceptions to the rules

There are facts that indicate histochemical staining with CR is non-specific. In particular, it has been shown that CR is not specific for amyloid and can also stain elastotic dermis, as well as hyaline deposits in colloid milium and in lipid proteinosis [179]. It was also found that CR can stain native proteins such as elastin [174–176] and collagen [61].

In medical practice, Fernandez-Flores [180] discovered that a heat artefact due to cautery in surgical specimens can cause false-positive results in the diagnosis of amyloidosis when staining biopsies with this dye.

False-positive results with CR also depend on salt concentration [137,181]. It was shown that the use of sodium chloride at 50% saturation led to strong staining of collagen and eosinophilic granules [181]. Sodium chloride saturation has been reported to restrict staining to ‘true’ amyloid, although collagen and elastic fibres can also show red fluorescence [136,181].

There are several parameters, such as choice of biopsy site, as well as staining and analysis of the tissue, that can affect the sensitivity and specificity of histochemical analysis [182]. Notably, fat pad biopsies are less invasive and reasonably have 73% sensitivity, but fat pad needle biopsies have sensitivities ranging from 58 to 85% [182]. The overall staining specificity lies within the range 75–100% for biopsies [182,183]. However, some papers report that fat pad aspirates stained with CR exhibited lower sensitivity [184]. Devata et al. [188] wrote: “this may be due to multiple variables including type of patient population, severity of disease with scant versus abundant amyloid, experience level of the interpreters, microscope type, polariser quality, room darkness, and time spent to detect amyloid” [189].

The inter-observer variability in interpretation of fat pad aspirates stained with CR was also reported [185–187]. Devata et al. [188] demonstrated that when detecting amyloid in abdominal fat pad aspirates in early amyloidosis, the polarised light microscopy method for CR-stained sections resulted in frequent false-negative results (9 out of 9, 100%). In addition, the investigators estimated the effects of inter-observer variability among four pathologists: sensitivity was 25–75% and specificity was 50–100% [188].

Interestingly, Klatskin [189] showed as early as 1969 that it is possible to reveal foci of green birefringence in connective liver tissue and several other organs in both humans and rats under appropriate processing conditions. Regrettably, early publications describing cases of amyloidosis with CR staining, lack a ‘materials and methods’ section. This makes it difficult to determine CR staining procedures, taking into consideration that staining conditions influence the result. Typically, assays are performed under extreme conditions with 50–80% ethanol, high salt and alkaline pH conditions for successful binding to amyloid [132]. Despite these extreme conditions, false-positive results associated with the binding of CR to collagen fibres and cytoskeletal proteins were obtained [61].

By 2012, it was shown that the sensitivity of CR staining for amyloidosis is related to the tissue source, specifically bone marrow (63%), kidney, liver and cardiac tissues (87–98%) and rectal (69–97%) tissues [182]. The difficulty is that sampling of biological material is invasive and a highly qualified and skilled surgeon is necessary to perform this task. Linke [60] also wrote: “CR-stained sections should be evaluated by an experienced observer and always using a positive control. An appropriate microscope, with a powerful light source, used in a dimmed room, is an essential requisite”.

The aforementioned sensitivity values for CR staining of amyloidosis in tissue sections are astonishing compared with the results of *in vitro* studies of amyloid aggregates/fibrils. It should be considered that amyloid plaques also contain non-proteinaceous components, including nucleic acids [190], lipids [191], metal ions [192] and glycosaminoglycans [193].

Despite all aforementioned challenges, CR stain apple-green birefringence under polarised light is the most popular method for detecting amyloid in tissue sections; however, it has limitations. It was shown, that CR stain by fluorescent microscopy significantly enhances the diagnostic yield [194]. CRF has been used since 1965 [59]. For instance, when slides of amyloidosis stained with CR were examined under UV light, the following results were obtained: ‘amyloid was coloured bright red, and other tissue structures were pale greenish grey’ [59]. In 2003, CRF was employed to determine cutaneous amyloidosis. The results obtained were as follows: ‘while the amyloid stained either weakly or very weakly with CR, while CRF was strong or intensive’ [159]. In 2010, while studying cutaneous amyloidosis, Fernandez-Flores reported that the results of his study [195] were not in agreement with those previously published [159]. For instance, colouration after the use of CRF was intense only when CR staining was pronounced [195]. In this paper, the author concluded that immunohistochemistry offers advantages over CR staining and CRF [195]. Some investigators suggest that sensitivity can be enhanced by combining CR staining with fluorescence and antibodies, since the classical CR method by itself is insensitive [60].

Results from *in vitro* studies on non-specificity of amyloid staining with CR are known [155,161,167]. In 1989, comparative studies on the binding of this dye to proteinaceous substances with various internal structures, including β-pleated sheets and α-helical conformations, were performed [161]. The researchers found that CR binds to both the β-pleated sheet conformation and the α-helical conformation of poly-l-lysine [161]. These authors also found that CR did not bind as well to either poly-l-serine or polyglycine as it did to insulin fibrils or β-poly-l-lysine, even though all of these homopolymers exist in the β-pleated sheet conformation [196]. It was also shown that CR can bind to native α-proteins, such as citrate synthase and interleukin-2 [168] and to recombinant α-proteins, such as recombinant human growth hormone and recombinant human interferon-α2b [197]. These findings indicate that the β-pleated sheet peptide backbone alone is insufficient to form the optimal structural substrate for CR binding. Considering the two negatively charged sulphonic acid groups in the dye molecule, it was suggested that CR binding depends on the content of charged amino acid groups in protein molecules [161]. In this regard, it became clear why peptides with no positively charged amino acid residues (other than at the N-terminus), such as poly-l-serine and polyglycine, possess very few CR binding sites and fail to induce a spectral shift, even though they have a β-pleated sheet conformation [161], and *vice versa*; an α-helical peptide with an abundance of positively charged residues, such as α-poly-l-lysine, binds well to CR [198,199]. Nevertheless, it should be noted that the presence of positively charged residues alone are not enough to promote CR binding, as evidenced by the binding of CR to native insulin [161]. The β-pleated sheet conformation is apparently necessary to orient cations into the optimal conformation for CR binding [161]. The authors pointed out that the binding of CR to β-poly-l-lysine was also pH dependent [161].

It is known that binding of CR to β-amyloid fibrils formed *in vitro* is dependent on рН and on the histidine residue content in the peptide molecules [200]. The Cegelski group, while exploring the *Escherichia coli* amyloid fibre curli using surface plasmon resonance, showed that CR binding is dependent on pH but is independent of the mutant curli fibres that lack histidine residues [201].

The difficulty of using CR to study amyloids lies in the fact that the dye can interfere with the processes of protein misfolding and aggregation [202,203]. In 1994, it was shown that CR and certain sulphated glycans are potent inhibitors of protease-resistant PrP accumulation in scrapie-infected cells [202]. Inhibition of huntingtin fibrillogenesis by CR was shown *in vitro* in 2000 [203]. Undoubtedly, these properties of CR may affect interpretation of the results.

In support of the fact that CR binds preferentially to β-sheets containing amyloid fibrils and can specifically inhibit oligomerisation and disrupt preformed oligomers, evidence for inhibition of the effect of CR on polyglutamine oligomerisation exists [204]. It has also been reported that, *in vitro*, CR inhibits the fibrillisation of β-amyloid [205], insulin [206] and amylin [207]. As a result of these findings, some researchers recommended employing CR, not for diagnosing, but for treating amyloidosis [208]. However, the anti-aggregation effect of CR is not observed for all amyloids. Evidence that this dye did not inhibit amyloid production *in vivo*, for instance are available in microbial systems such as *E. coli* and *Salmonella* [201].

Notably, by 2001, the question ‘Is CR an amyloid-specific dye?’ had already been raised in scientific literature [167]. Researchers studied the structural specificity of CR binding to amyloid fibrils using an induced CD assay [167]. The authors found that the native conformations of insulin and Ig light chain induced CR circular dichroism, but with spectral shapes that differed from those of fibrils of the same proteins [167]. Khurana et al. [167] also showed that a wide variety of native proteins exhibited induced CR circular dichroism, indicating that CR bound to representative proteins from different secondary structure classes, such as α (citrate synthase), α+β (lysozyme), β (concavalin A) and parallel β-helical proteins (pectate lyase) [167]. These scientists also demonstrated that CR induced oligomerisation of native proteins using small angle X-ray scattering and cross-linking analysis [167].

Researchers have also tested the feasibility of using CR to detect amyloids in tissue sections [167]. The results of this study showed that CR binds to many native proteins and is not specific to secondary structure. These findings explain the false-positive results obtained on cytoskeletal proteins that are stable under the conditions used for CR staining in tissue sections [167].

**Figure 2:**
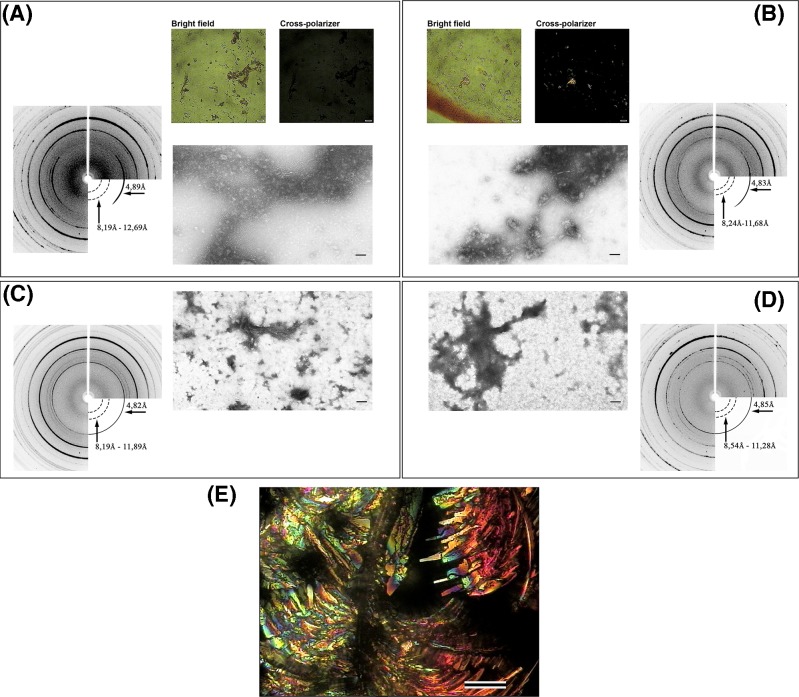
Investigation of SMT aggregates (**A**) SMT(KCl) aggregates: X-ray diffraction (left); CR polarisation microscopy of the aggregates, scale: 1 μm (top right); electron microscopy of negatively stained aggregates, scale: 100 nm (bottom right). (**B**) SMT(Gly) aggregates: CR polarisation microscopy of the aggregates, scale: 1 μm (top left); electron microscopy of negatively stained aggregates, scale: 100 nm (bottom left); X-ray diffraction (right). (**C**) SMT(KCl) aggregates after partial disaggregation: X-ray diffraction (left), electron microscopy of negatively stained titin aggregates, scale: 100 nm (right). (**D**) SMT(Gly) aggregates after partial disaggregation: electron microscopy of negatively stained aggregates, scale: 100 nm (left); X-ray diffraction (right). (**E**) Microscopy under polarised light of a dried drop of buffer containing 0.15 M glycine-KOH, pH 7.2–7.4 and CR, scale: 100 μm. For electron microscopy, 2% aqueous uranyl acetate staining was used.

The non-specificity of CR as an amyloid dye was also shown in our *in vitro* studies [31]. We found that smooth muscle titin (SMT) from chicken gizzards can form amorphous aggregates in two different solutions containing: (i) 0.2 M KCl, 10 mM imidazole, pH 7.0 (SMT(KCl) aggregates) (Figure 2A) and (ii) 0.15 M glycine-KOH, pH 7.2–7.4 (SMT(Gly) aggregates) (Figure 2B). The amyloid natures of SMT(KCl) and SMT(Gly) aggregates were confirmed by X-ray diffraction [31]. CR-stained SMT(Gly) aggregates had ‘yellow to apple-green birefringence under polarised light’, while no such birefringence for SMT(KCl) aggregates was observed (Figure 2A,B). Interestingly, SMT(KCl) and SMT(Gly) aggregates, dialysed against a solution containing 0.6 M KCl, 30 mM KH_2_PO_4_, 1 mM DTT and 0.1 M NaN_3_ at pH 7.0, also had amyloid X-ray patterns (Figure 2C,D). In both cases, ‘yellow to apple-green birefringence under polarised light’ was absent [31]. In our *in vitro* study, we did not observe a characteristic shift in the CR maximum optical absorbance from 490 to 540 nm in any sample, indicating the binding of the dye to titin amyloids [31].

Note that a dried buffer (0.15 M glycine-KOH, pH 7.2–7.4) containing CR without protein had yellow to apple-green birefringence under polarised light along with other colours (Figure 2E). In our experiments, it was apparent that differences found in titin aggregates stained with CR were associated with the buffer (despite performing a water wash) and not with the amyloid nature of the protein aggregates.

## Conclusion

CR, now a classic dye, has played a role in the history of amyloid research. However, there is currently ample evidence of nonspecific binding of CR in studies on the identification of amyloids. The dye has been shown to bind non-amyloid substances, in addition to not binding amyloids in *in vitro* experiments and in *in vivo* histochemical studies. There is evidence that a specific amyloid cross-β structure is not necessary for binding to the dye, and the mechanism of CR binding to amyloids depends on a number of conditions, including the type of solvent, the composition of the solution, pH etc.

Due to modern scientific and methodological developments, researchers have a variety of possible methods available to study amyloids, according to their interest (Table 1) and can take into account the characteristic features and limitations of different methods. In this review, we discussed in detail the disadvantages of the CR staining method. Care should be taken when using the CR staining method to avoid misinterpretation of data.

Nevertheless, this technique has some merits. It is quick and easy to stain amyloids *in vitro*. CR can be employed for amyloid research in studies that utilise appropriate control samples (e.g., buffer alone and a sample of amyloid precursor) and other additional techniques to detect or characterise amyloid aggregates/fibrils.

Notwithstanding the imperfect specificity of CR binding to amyloids, the dye can be used in histochemical studies. It should be considered that positively stained tissue elements, such as elastin, collagen, eosinophilic granules and others, can usually be identified by their appearance or location. Moreover, when amyloidosis is suspected, in the presence of negative results obtained after CR staining, it is necessary to use immunohistochemistry for confirmation. Researchers must also remember that green is not the only colour that can be observed in the CR birefringence assay. Lastly, the use of CR alone is insufficient for diagnosing amyloidosis.

In conclusion, this historical analysis shows that the relationship between CR and amyloids is more complex than it appears initially. We anticipate that this review will provide insight into the expected outcomes following the use of CR.

## Abbreviations

CR: Congo red
CRI: CR index
CRF: CR fluorescence
SMT: smooth muscle titin
SMT(KCl) aggregate: aggregate of SMT in solution containing 0.2 M KCl, 10 mM imidazole, pH 7.0
SMT(Gly) aggregate: aggregate of SMT in solution containing 0.15 M glycine-KOH, pH 7.2–7.4
